# Footprints of domestication revealed by RAD-tag resequencing in loquat: SNP data reveals a non-significant domestication bottleneck and a single domestication event

**DOI:** 10.1186/s12864-017-3738-y

**Published:** 2017-05-06

**Authors:** Yunsheng Wang, Muhammad Qasim Shahid, Shunquan Lin, Chengjie Chen, Chen Hu

**Affiliations:** 1 Key Laboratory of Biology and Germplasm Creation of Horticultural Crop (Southern China), Ministry of Agriculture, South China Agriculrual University, Guangzhou, 510642 China; 2 College of Environment and Life Science, Kaili University, Guizhou, 556011 China

**Keywords:** *Eriobotrya japonica*, Population genomics, Sequencing, SNP marker, Wild loquat

## Abstract

**Background:**

The process of crop domestication has long been a major area of research to gain insights into the history of human civilization and to understand the process of evolution. Loquat (*Eriobotrya japonica* Lindl.) is one of the typical subtropical fruit trees, which was domesticated in China at least 2000 years ago. In the present study, we re-sequenced the genome of nine wild loquat accessions collected from wide geographical range and 10 representative cultivated loquat cultivars by using RAD-tag tacit to exploit the molecular footprints of domestication.

**Results:**

We obtained 26.4 Gb clean sequencing data from 19 loquat accessions, with an average of 32.64 M reads per genotype. We identified more than 80,000 SNPs distributed throughout the loquat genome. The SNP density and numbers were slightly higher in the wild loquat populations than that in the cultivated populations. All cultivars were clustered together by structure, phylogenetic and PCA analyses.

**Conclusion:**

The modern loquat cultivars have experienced a non-significant genetic bottleneck during domestication, and originated from a single domesticated event. Moreover, our study revealed that Hubei province of China is probably the origin center of cultivated loquat.

**Electronic supplementary material:**

The online version of this article (doi:10.1186/s12864-017-3738-y) contains supplementary material, which is available to authorized users.

## Background

The domestication of plant and animal is the most important development in the past 13,000 years of human history [1]. The studies on the genetic mechanisms of crop domestication help us to understand the establishment/origin of cultivated species and the history of human civilization, but also offer comprehensive utilization of wild resources to improve the existing varieties and create new germplasm [1–3]. Hence, crop domestication is a very popular topic, and multidisciplinary research methodologies have been used to evaluate domestication [1, 3–5]. In recent decade, research related to crop domestication has been transformed by technologies and discoveries in the genome sciences as well as information-related sciences that are providing new ways for bioinformatics and systems biology [6, 7].

There are six to eight major origin centers of crops in the world and China is the most important one [8, 9]. In addition to the major crops, such as rice [10], soybean [11] and some other grain crops and vegetables, there are about 52 kinds of fruit crops that were domesticated in China [12]. Among them, the loquat (*Eriobotrya japonica* Lindl.) is one of the representative subtropical evergreen fruit trees [13]. Loquat is a delicious fruit, rich in amino acid, carbohydrate, fat, cellulose, pectin, carotene, tannin, organic acid, vitamin A, B, C, B1 and B2, calcium, potassium, phosphorus, iron and other vital elements [14, 15]. Loquat is also a kind of important traditional Chinese herbal medicine and various parts such as root, stem, leaf, flower and fruit of loquat tree can be used as a medicine for the normal functioning of lungs, arresting cough, anti-inflammatory and strengthening of stomach [16], and also containing some anti-cancer compounds [17, 18].

According to the “historical records”, one of the greatest books in the Chinese history, loquat trees had been cultivated in the royal gardens for the refreshment of king and princess in the Han Dynasty of China 2000 years ago. In twelfth century, loquat had been introduced into Japan, and then from China or Japan to the rest of the world [13]. Today, there are more than 30 countries planting loquat in the world, and China producing about 80% loquat of the world, with the annual production of about 1 million tons [19]. In recent years, loquat planting area and yield showed increasing trends because of high economic returns. Many studies on loquat had been executed, including biology [20], molecular phylogeny [21, 22], genetic diversity analysis [23–26], breeding [27], physiology [28, 29], pharmacology [17, 18], and molecular biology [30]. However, domestication of loquat at molecular level has not been discussed.

Single nucleotide polymorphism (SNP) originated from single nucleotide substitute mutation or insertion/deletion, and it is the most abundant type of variation in the species or genomes [31], and have many utilizations in the field of life sciences, such as molecular genetics, molecular ecology, evolutionary genetics and association analysis [32–35]. In recent years, with the development of high-throughput genome sequencing technologies and the progress in bioinformatics analyses, the whole genome of number of species have been sequenced and re-sequenced. One of the most important achievements of these sequencing works is that SNP markers of these species have been exploited and were used to construct the genetic map for studies of population genomics, phylogeography, ecological genomics and genome-wide association study (GWAS) [36–39].

As for non-model plant without reference genome, new ways of high throughout-sequencing have also been created to exploit SNPs at large scale, and the restriction-site associated DNA tags (RAD) is a famous one. The so-called “Restriction-site Associated DNA” (RAD) method was first described by Miller et al. [40]. The concept is based on acquiring the sequence adjacent to a set of particular restriction enzyme recognition sites. The application of high throughput sequencing technology has allowed significant progress in developing a RAD genotyping platform [41]. Specifically, large volumes of polymorphism data can be generated by applying massive parallel sequencing and multiplexing with RAD tag libraries [42], which make it widely used technology in population genetic studies [43–47].

In this study, we used RAD-tag tacit sequencing to genotype 10 representative loquat cultivars and nine wild loquat accessions, which collected from different natural habitats. The main aim was to detect the SNPs for whole loquat genome and to explore domestication event of cultivated loquat, such as the geographical origin of cultivated loquat, whether cultivated loquat population is originated from a single- or multi-domesticated events, and whether genetic bottleneck appeared as did in the other crops during domestication.

## Results

### Sequencing data

We obtained a total of 26.4 Gb clean sequencing data from 620.2 M reads of Illumina Solexa sequencing. The reads of each sample ranged from 14.16 M to 95.07 M, with an average of 32.64 M reads. The sequence data of each sample was ranged from 608.68 to 3898.03Mb, with an average sequence data of 1390 Mb, and the average read length was 42.56 bases. The quantity and quality of sequencing data obtained from wild groups were lower than that from cultivated groups under the same experimental conditions. However, the sequencing quality scores of 20 (Q20), which represent an error rate of 1 in 100, with a corresponding call accuracy of 99%, of sequencing data of all samples were more than 98%, indicating that the sequencing was of high quality, and the data quantity and quality fulfill the requirements for population genomics analysis. The mean GC content of sequencing data was 35.12%, which was slightly higher in wild group (35.35%) than the cultivated group (34.92%), suggesting that the sequences adjacent to *Eco*RI restriction enzyme sites had low content of GC sequence (Table 1).

**Table 1:** Summary of the raw data obtained by RAD-tag resequencing

Group	Genotype ID	Raw data			
		Reads (M)	Bases (Mb)	GC (%)	Q20 (%)
Wild	TS_W	23.92	1028.62	35.49	99.01
	WF_W	14.16	608.68	35.15	98.55
	BD_W	32.03	1357.43	35.86	99.21
	LC_W	16.11	676.79	35.01	98.91
	SM_W	17.91	788.21	35.25	98.49
	MT_W	18.08	741.38	36.96	98.77
	BJ_W	33.70	1381.59	34.66	98.66
	AL_W	33.19	1460.43	34.98	99.01
	HZ_W	57.80	2369.91	34.76	98.95
	Average	27.43	1157.00	35.35	98.84
Cultivated	DWX_C	24.82	1067.40	34.96	98.89
	BY_C	34.57	1486.72	35.09	99.09
	YN_C	32.03	1441.15	35.03	99.11
	SJPP_C	23.76	1021.64	34.97	99.14
	RTBS_C	67.82	2994.30	34.72	98.20
	ZZ6H_C	23.42	1030.38	35.00	99.17
	MM_C	95.07	3898.03	34.68	98.90
	MBC_C	27.61	1214.65	35.06	99.13
	Biano_C	23.10	947.06	34.79	99.08
	Algeria_C	21.10	886.33	34.90	99.19
	Average	37.33	1598.77	34.92	98.99
Total Mean		32.64	1389.51	35.12	98.92

See Table 6 for genotypes detail

### SNP calling and its distribution pattern

Beside only 2 Mb assembled sequence data of a wild loquat accession, BD_W, which was collected from Badong county of Hubei province, we obtained about 6–7 Mb assembled sequences for each genotype from other 18 loquat genotypes. We called SNP site according to the following fundamental principle: the homozygous site assembled by at least 10 loquat accessions were sequenced and at least a base variant was found, and we identified a genotype set with 86454 SNPs by this method (Additional file 1: Table S1). We detected about 60000–70000 SNPs in each loquat accession, except about 20000SNPs found in BD_W sample. The distribution density of SNP in the genome of 19 samples was from 8.24 to 10.52 SNPs/Kb (Table 2), and the average density in wild loquat was higher in wild population than did in cultivated population (i.e. total SNPs per kb: 10.16 vs 10.04 (2.4%), heterozygous SNPs per Kb: 1.99 vs 2.01 (-1.0%), and homozygous SNPs per Kb: 8.16 vs 7.93 (2.9%) were detected in wild and cultivated populations, respectively)). However, *t*-test of independent samples on the frequency of total number of SNPs, homozygous SNPs and heterozygous SNPs exhibited a non-significant differences between cultivated and wild loquat groups (P > 0.05). We further obtained a SNP data set composed of 6406 SNPs with no gap from whole loquat population (Additional file 1: Table S2). Of these, 4807 and 1599 sites were SNPs and fixed in wild loquat population, while 4231 and 2175 sites were SNPs and fixed in cultivated loquat population, respectively. The ratio of total number of SNPs, with no gap, was 13.61% higher in wild loquat population than cultivated loquat population (Table 3).

**Table 2:** Summary of the SNPs with a genotyping rate of homozygous sites less than 50%

Group	Genotype ID	Assembled sequence (bp)	Total SNPs	Heterozygous SNPs	Homozygous SNPs	Total SNPs per kb	Heterozygous SNPs number per Kb	Homozygous SNPs per Kb
Wild	AL_W	7645931	77745	15124	62621	10.17	1.98	8.19
	BD_W	2107974	20324	2441	17883	9.64	1.16	8.48
	BJ_W	6828806	71837	13911	57926	10.52	2.04	8.48
	HZ_W	7063593	72665	12810	59855	10.29	1.81	8.47
	LC_W	6755448	69772	14319	55453	10.33	2.12	8.21
	MT_W	6693879	69792	15421	54371	10.43	2.30	8.12
	SM_W	7406774	72054	18028	54026	9.73	2.43	7.29
	TS_W	7802112	78502	16920	61582	10.06	2.17	7.89
	WF_W	7105355	72840	13639	59201	10.25	1.92	8.33
	Average	6601097	672812	13624	53658	10.16	1.99	8.16
Cultivated	Algeria_C	6734550	69033	17950	51083	10.25	2.67	7.59
	Biano_C	6378374	65886	13780	52106	10.33	2.16	8.17
	BY_C	7509796	76407	15521	60886	10.17	2.07	8.11
	DWX_C	7191808	74136	13487	60649	10.31	1.88	8.43
	MBC_C	7473459	74392	17565	56827	9.95	2.35	7.60
	MM_C	7170711	73359	18597	54762	10.23	2.60	7.64
	RTBS_C	6740816	55532	2993	52539	8.24	0.44	7.79
	SJPP_C	7199676	73112	15336	57776	10.15	2.13	8.02
	YN_C	7668847	75122	13710	61412	9.80	1.79	8.01
	ZZ6H_C	7310710	72836	14474	58362	9.96	1.98	7.98
	Average	7137875	70982	14341	56640	9.94	2.01	7.93
Total	Mean	6883612	69229	14001	55227	10.04	2.00	8.04

See Table 6 for genotypes detail

**Table 3:** Summary of SNPs from the genotyping data of all 19 loquat accessions with no gap

Data ID	Number of total SNPs	Number of fixed sites
Whole loquat population	6406	0
Wild population	4807	1599
Cultivated population	4231	2175

### Population genetic structure and genetic differentiation

AMOVA analysis of 19 loquat genotypes, based on the SNP data with no gap, revealed that the variance components among populations, among individuals within populations, and within individuals were 14.45%, 25.62%, and 59.93% of the total genetic variance, respectively (Table 4). The results showed that the heterozygosity within loquat individuals accounted for most of the genetic diversity of whole loquat population. The pairwise fixation index, *F*_ST_, was 0.16, which was estimated from pairwise comparison between wild and cultivated groups and was significantly different from zero (*P* < 0.001). These results showed that highly significant genetic differentiation happened between wild and cultivated loquat populations (Table 5).

**Table 4:** Analysis of molecular variance (AMOVA) of 19 loquat genotypes

Source of variation	Sum of squares	Variance components	Percentage variation
Among populations	3347.063	125.63590	14.44924
Within populations	16432.094	222.73111	25.61604
Within individuals	9901.500	521.13158	59.93472
Total	29680.658	869.49858

**Table 5:** Genetic differentiation between wild and cultivated populations

Pairwise index (*F*_ST_)	Wild population	Cultivated population
Wild population	-	***
Cultivated population	0.15955	-

### Phylogenetic analysis

**Fig. 1:**
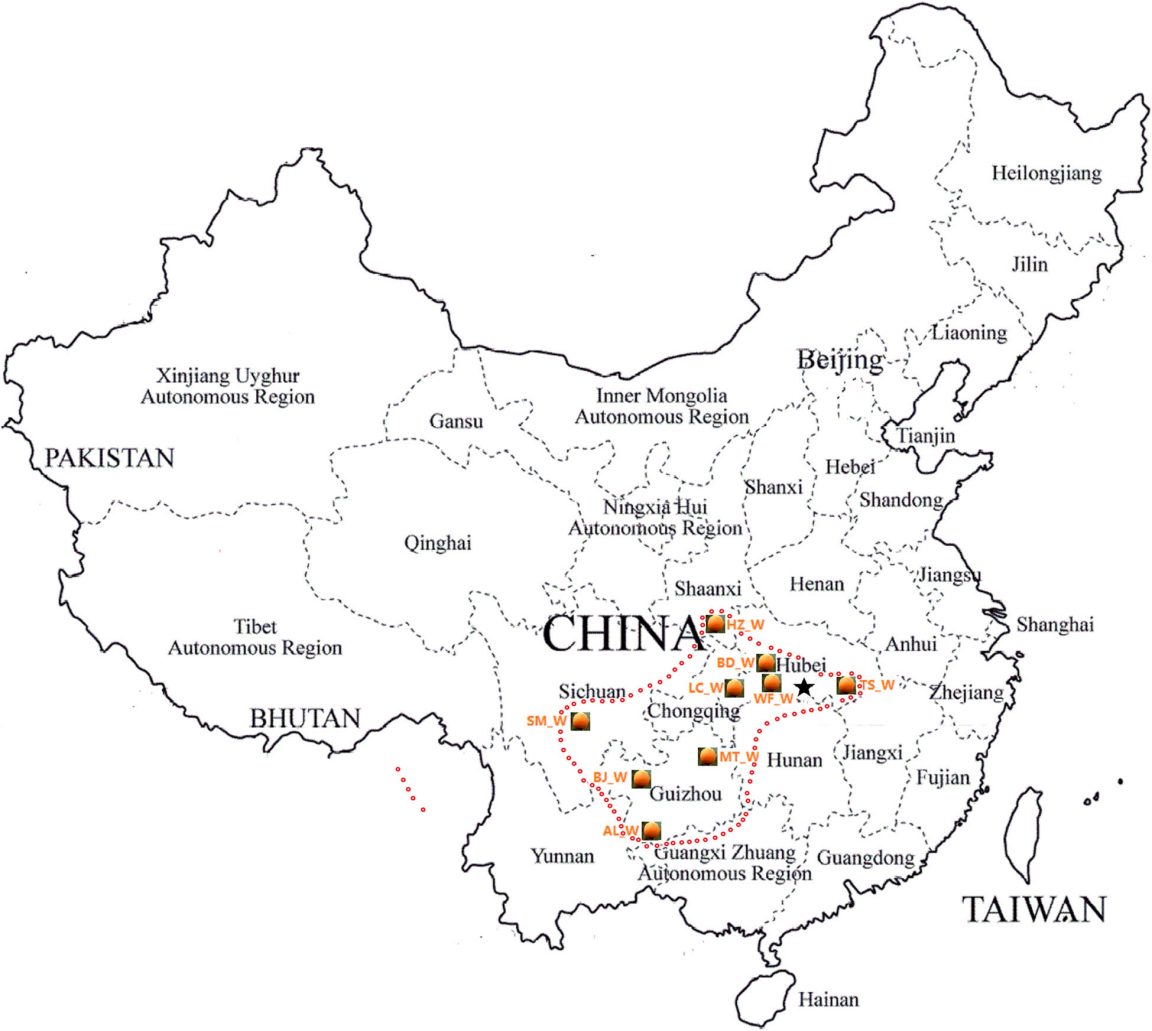
Phylogenetic tree of 19 loquat genotypes was constructed based on neighbor-joining (bootstrap value =500) method. AL_W, BJ_W, MT_W, SM_W, HZ_W, BD_W, LC_W, WF_W, TS_W represent the wild loquat genotypes; AL_W, BJ_W and MT_W were collected from Anlong, Bijie and Meitan counties of Guizhou province, SM_W was collected from Simian county of Sichuan province, HZ_W was collected from Hanzhong county of Shanaxi province, BD_W, LC_W, WF_W, TS_W were collected from Badong, Lichuan, Wufeng and Tongshan counties of Hubei province, respectively; DWX_C, BY_C, YN_C, SJPP_C, RTBS_C, ZZ6H_C, MM_C, MBC_C, Biano_C, Algeria_C are all loquat cultivars, and known as Dawuxing, Baiyu, Yunan, Sijipipa, Ruantiaobaisha, Zaozhong No.6, Mogi, MBC, Biano and Algeria, respectively

Unrooted phylogenetic tree of 19 samples showed that nine samples of wild group clustered together at one end of the tree, and 10 cultivated samples were clustered at the other end of the tree (Fig. 1). In the wild group, three samples from Bijie county, Anlong county, and Meitan county, Guizhou province clustered into close proximity to each other, and the sample from Shimian county, Sichuan province was also close to the aforementioned accessions, indicating that the genetic distance of wild loquat population had some regional relationships. However, the wild samples from Hubei and Shaanxi province were more close to cultivated loquat samples and the closest one was TS_W. Interestingly, the cultivar, Sijipipa, exhibited the closest relationships with the wild loquat population, and the cultivars Dawuxing, Yunan and Biano were clustered far from the wild loquat population.

### PCA analysis

**Fig. 2:**
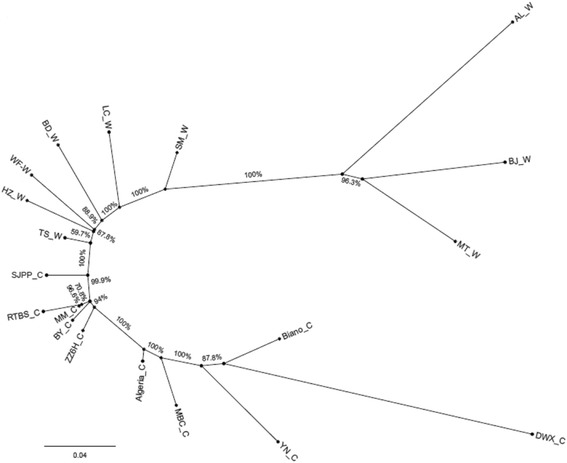
Principal component analysis (PCA) of 19 loquat genotypes. AL_W, BJ_W, MT_W, SM_W, HZ_W, BD_W, LC_W, WF_W, TS_W represent the wild loquat genotypes; AL_W, BJ_W and MT_W were collected from Anlong, Bijie and Meitan counties of Guizhou province, SM_W was collected from Simian county of Sichuan province, HZ_W was collected from Hanzhong county of Shanaxi province, BD_W, LC_W, WF_W, TS_W were collected from Badong, Lichuan, Wufeng and Tongshan counties of Hubei province, respectively; DWX_C, BY_C, YN_C, SJPP_C, RTBS_C, ZZ6H_C, MM_C, MBC_C, Biano_C, Algeria_C are all loquat cultivars, and known as Dawuxing, Baiyu, Yunan, Sijipipa, Ruantiaobaisha, Zaozhong No.6, Mogi, MBC, Biano and Algeria, respectively

The results of principal components analysis of the 19 genotypes showed that the first principal component could explain 5.09% genetic variation of whole loquat population, and the second principal components can explain 3.38% genetic variation of whole loquat population (Fig. 2). The first and the second principal components could clearly separate wild and cultivated loquats, and the results showed that the wild and cultivated loquat have entirely different evolutionary trends.

### Population structure

**Fig. 3:**
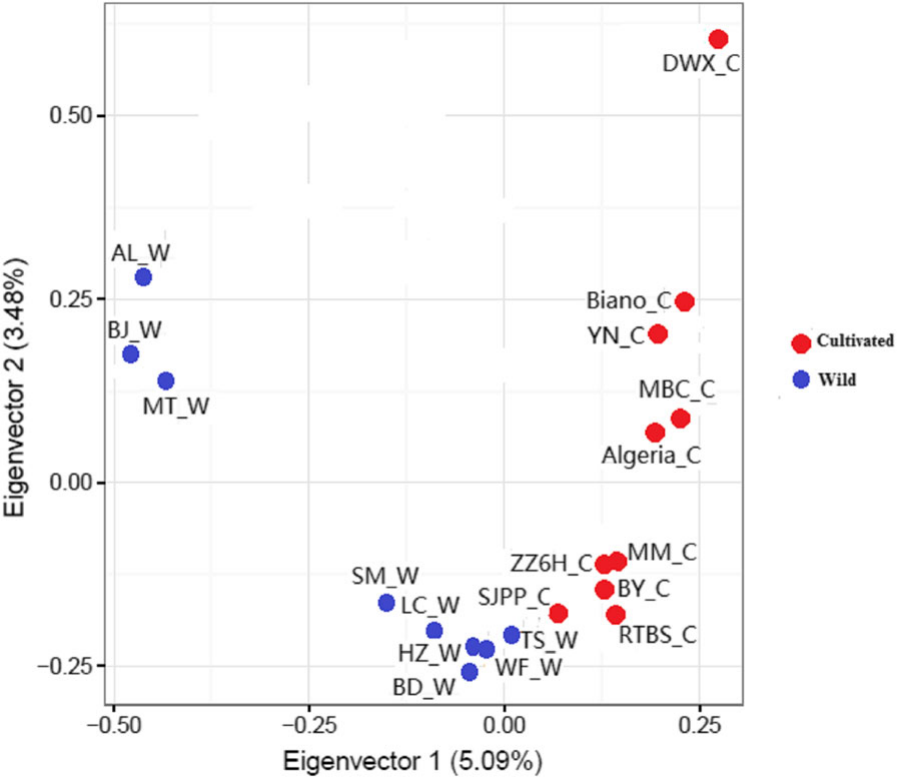
Population structure analysis of 19 loquat genotypes. X- and Y-axis are representing genotypes and probability levels, respectively. AL_W, BJ_W, MT_W, SM_W, HZ_W, BD_W, LC_W, WF_W, TS_W represent the wild loquat genotypes; AL_W, BJ_W and MT_W were collected from Anlong, Bijie and Meitan counties of Guizhou province, SM_W was collected from Simian county of Sichuan province, HZ_W was collected from Hanzhong county of Shanaxi province, BD_W, LC_W, WF_W, TS_W were collected from Badong, Lichuan, Wufeng and Tongshan counties of Hubei province, respectively; DWX_C, BY_C, YN_C, SJPP_C, RTBS_C, ZZ6H_C, MM_C, MBC_C, Biano_C, Algeria_C are all loquat cultivars, and known as Dawuxing, Baiyu, Yunan, Sijipipa, Ruantiaobaisha, Zaozhong No.6, Mogi, MBC, Biano and Algeria, respectively

Population structure helps us to understand the evolutionary process of a population or species through the association between genotype and phenotype, and to determine the groups or subgroups of different individuals/populations. When k was set as 2, Al_W, MT_W, BJ_W, SM_W, LC_W and BD_W were clustered into one wild group, and nine cultivated loquat cultivars were clustered into cultivated group, and HZ_W, WF_W, TS_W and SJPP_C were assigned to aforementioned two groups. However, HZ_W, WF_W and TS_W have a higher probability to be included in wild group, and the SJPP_C has a higher probability to join cultivated group. When k was set as 3, Al_W, MT_W and BJ_W from Guizhou province constituted a group, MBC_C, Biano_C, YN_C and DWX_C were clustered into one group, and BD_W, HZ_W, WF_W TS_W and SJPP_C were assembled into another group, while SM_W, LC_W, BY_C, ZZ6H_C, MM_C and Algeria_C were clustered into two separate groups. When k was set as 4, three samples collected from Guizhou province were further divided into two groups, and the clustering pattern of other samples was almost consistent with K = 3; When k was set as 5, the wild samples were divided into three groups with three samples in each group, and the cultivated loquat cultivars was clustered into two groups (Fig. 3).

## Discussion

### The representativeness of samples

According to the extensive survey, the wild loquat (*Eriobotrya japonica* Lindl.) populations have been found in Hubei, Sichuan, Yunnan, Guizhou, Guangxi and Guangdong provinces of China. Among them, Hubei and Guizhou provinces are the major distributing areas of wild loquat [48]. In the present study, a total of 9 wild accessions and 10 loquat cultivars were selected for sequencing. Among 9 wild loquat samples, four samples were collected from different regions of Hubei province, three samples from different regions of Guizhou province, one from Sichuan province, and one from Shanxi province. All these nine samples were regional representatives of species distribution range and separated from each other by far geographical distance. Among 10 loquat cultivars, six are the main cultivars of different loquat producing areas of China, such as Zhejiang province, Fujian province, Jiangsu province, Sichuan province, Yunnan province and Guangdong province, and other four are the main cultivars of Japan, Italy, Spain and the United States of America. Molecular pedigree analysis showed that those cultivars almost represent different genetic clusters of current loquat cultivars around the world [25, 26].

### Genetic bottleneck in cultivated loquat during domestication

In the process of crop domestication, the genetic bottleneck is common, which indicate that the effective size of crops population were significantly fewer than its corresponding wild progenitor population [49]. Compared to the nearest wild progenitors, crops population appeared to have lower genetic diversity, higher level of linkage disequilibrium and lower SNP frequencies, such as rice [38, 39], soybean [11, 50], cucumber [51], peach [52], and tomato [53]. However, there was no clear genetic bottleneck in several crops, such as grape [54]. In this study, we found that the SNP frequencies in wild loquat population were only 2.4% higher than that in cultivated loquat population. Meanwhile, AMOVA analysis showed that 85.55% of genetic variants existed among- and inter-individuals, while only 14.45% happened between wild and cultivated populations. The independent samples *t*-test exhibited non-significant differences between cultivated and wild loquat groups (*P* > 0.05) for the frequency of total number of SNPs, homozygous SNPs and heterozygous SNPs. Above all results suggested that a non-significant genetic bottleneck appeared during the domestication of loquat. We deduced following major reasons for these results: 1) the domestication history of loquat is not so long; 2) loquat domestication is not yet complete, because there are non-significant differences for physiological and morphological traits between cultivated and wild loquat except that the fruit size of cultivated loquat is bigger than that of wild loquat.

### The origin center of cultivated loquat

Discovering the origin center of a crop is not only an interesting scientific question, but also an important social science issue, because the ancient civilization of mankind is actually a farming civilization, and the action of crops domestication represents the height of the agricultural civilization to a certain extent. In fact, most crops were thought to have originated in the more developed areas of ancient civilizations [2, 9]. The valley of Qingshuijiang river of South-West Hubei province or valley of Daduihe river of South-West Sichuan province have been inferred as the original place of cultivated loquat according to the field investigations of morphological traits and geographical distribution [55, 56]. In this study, the sample, TS_W, collected from Hubei province was the most closest to the cultivated loquat according to the phylogenetic tree. Structure analysis also showed that this genotype was clustered with some cultivated loquat cultivars, such as SJPP_C (K = 2, 3, and 4). However, the wild sample, SM_W from Sichuan province, was far from the cultivated loquat based on above mentioned analysis. According to the archaeological work, in the 1970s, when people dug an ancient tomb that was constructed in Han dynasty, which is located in Jiangling county of Hubei province and near to Tongshan county, the carbonization relic of loquat seed was found among many funeral items, and this was the earliest archaeological evidence of loquat cultivation [13]. So, based on molecular and archaeological evidence, we deduced that the Hubei province, not Sichuan province, might be the origin center of cultivated loquat.

### A single domestication origin of cultivated loquat

A crop that comes from a single domestication event or multiple domestication events is often present dispute based on the ancient book record and archaeology, which was used in corresponding research in the past [57–59]. However, modern molecular phylogeny, molecular population genetics and phylogeographical studies showed that most crops were evolved from single domesticated event, such as einkorn wheat [60], potato [61], soybean [50], maize [62], *japonica* rice [10], and cucumber [63]. Multi-domesticated events were not so common and it was observed in common bean [64] and barley [65].

China has about 2000 years long history for loquat cultivation until the twelfth century. Loquat was introduced into Japan from China and then from China and Japan to other parts of the world [19]. In the process of artificial selection and ecological adaptability, cultivated loquats differentiated into different cultivars or strains. Liu et al. [66] revealed three groups of cultivated loquat by analyzing 100 morphological and biochemical traits. Cultivated loquat of China was divided into two categories, namely as subtropical and tropical groups according to the regional ecological adaptability [67]. Two ecological groups of cultivated loquat were found according to the plant morphology such as leaf color, fruit size, and sugar content [48]. These analyses suggested that cultivated loquat may have been domesticated at multiple places, or may have been domesticated at just one place, and then spread outside along different routes, and differentiated into different groups. In the present study, structure, phylogenetic and PCA analyses have shown that all the cultivated loquat samples were clustered into the same group, which showed that cultivated loquat samples exhibited mono-phylogenic origin. So, we concluded that cultivated loquat was originated from a single domestication event. However, cultivated loquat cultivars exhibited differentiation by structure analysis (k = 5). One group, SJPP_C, RTBS_C, BY_C, ZZ6H_C and MM_C, might be comprised of early domesticated cultivars, and another group involving Biano_C, YN_C and DWX_C might be domesticated recently, while Algeria_C and MBC_C clustered between these two groups.

## Conclusions

Here, we re-sequenced the genome of nine wild loquat accessions collected from wide geographical range and 10 representative cultivated loquat cultivars by using RAD-tag tacit to exploit the molecular footprints of domestication. The results showed that the SNP density and numbers were non-significantly higher in the wild loquat populations than that in the cultivated populations. All cultivars were clustered together by structure, phylogenetic and PCA analyses. The modern loquat cultivars have experienced a non-significant genetic bottleneck during domestication, and originated from a single domesticated event. Moreover, our results revealed that Hubei province of China might be the origin center of cultivated loquat.

## Methods

### Plant sampling and DNA extraction

**Fig. 4:**
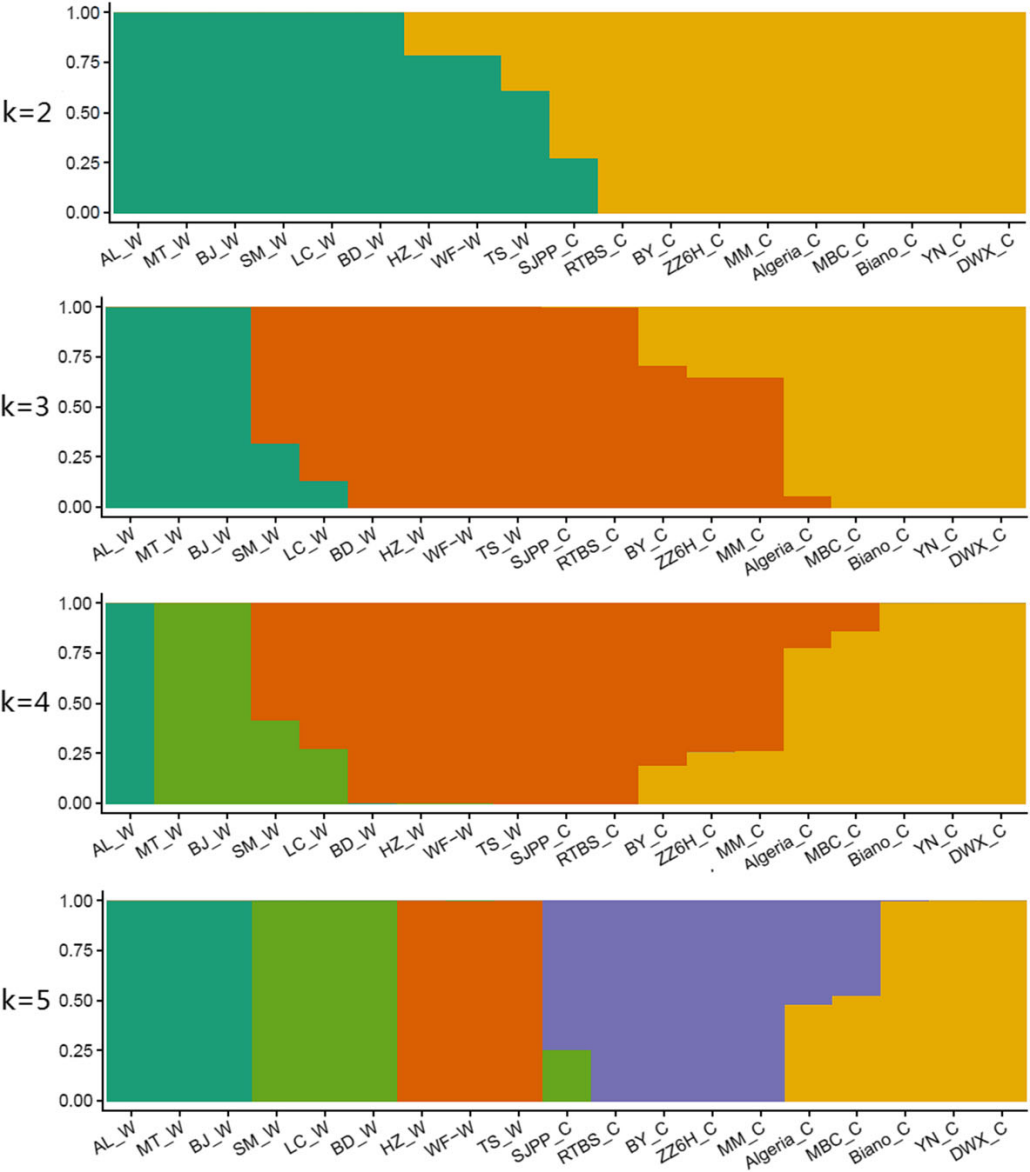
The sampling location of wild loquat genotypes. Orange levels (loquat fruit) represent the sampling sites of wild loquat genotypes, star (black) represents the position of earliest archaeological site where carbonation relic of loquat seed was found, and the area inside the red circles is natural habitat of wild loquat. AL_W, BJ_W and MT_W were collected from Anlong, Bijie and Meitan counties of Guizhou province, SM_W was collected from Simian county of Sichuan province, HZ_W was collected from Hanzhong county of Shanaxi province, BD_W, LC_W, WF_W, TS_W were collected from Badong, Lichuan, Wufeng and Tongshan counties of Hubei province, respectively

The leaves of ten cultivated loquat cultivars were sampled from Horticultural germplasm conversation center of South China Agricultural University (SCAU), where most of the major cultivated loquat cultivars of the world have been planted, and nine wild loquats were sampled from native wild loquat populations representing their natural distribution range (Table 6; Fig. 4). The DNA was extracted according to CTAB method [68].

**Table 6:** Names and geographical origin of loquat genotypes used in the study

Code	Genotype	Geographical origin
DWX_C	Dawuxing	Sichuan province, China
BY_C	Baiyu	Jiangsu province, China
YN_C	Younan	Guangdong province, China
SJPP_C	Sijipipa	Yunnan province, China
RTBS_C	Ruantiao baisha	Zhejiang province, China
ZZ6H_C	Zaozhong No.6	Fujian province
Biano_C	Biano	Italy
MBC_C	MBC	America
MM_C	Mogi	Japan
Algeria_C	Algeria	Spain
TS_W	Wild loquat	Tongshan county, Hubei province
WF-W	Wild loquat	Wufeng county, Hubei province
BD_W	Wild loquat	Badong county, Hubei province
LC_W	Wild loquat	Lichuan county, Hubei province
MT_W	Wild loquat	Meitan county, Guizhou province
BJ_W	Wild loquat	Bijie county, Guizhou province
AL_W	Wild loquat	Anlong county, Guizhou province
SM_W	Wild loquat	Shimian county, Sichuan province
HZ_W	Wild loquat	Luding county, Sichuan province

### The construction of RAD-tag libraries

Mixed library of RAD-tag (restriction-site associated DNA tags) was constructed in this study by the protocols described by Etter et al. [69], and it is as follows: (A) Restriction enzyme *Eco*RI digested about 1μg genomic DNA and joint P1 adapter ((P1-FOR-xxxxx: ′ -Phos- AATGATACGGCGACCACCGAGATCTACACTTTCCCTACACGACGCTCTTCCGATCTXxxxxTGC*A-3 ′ P1-REV-xxxxx: 5 ′ -Phos-xxxxxAGATCGGAAGAGCGTCGTGTAGGGAAAGAGTGTAGA; TCTCGGTGGTCGCCGTATCAT*T-3 ′) which contains sequences of primers P1-PCR (5 ′-AATGATACGGCCCACCGA-3 ′) for amplification, primer binding sites in Illumina Genome Analyzer and short tags to distinguish samples; (B) Samples with different adapters were mixed to be broken into segments of 300 ~ 700bp using physical method; (C) Joint P2 adapter (P2-FOR:′ -Phos-GATCGGAAGAGCGGTTCAGCAGGAATGCCGAGACCGATCAGAACAA-3 ′P2-PE-REV: 5′ -Phos CAAG CAGAAGACGGCATACGAGATCGGTCTCGGCATTCCTGCTGAACCGCTCTTCCG ATC*T-3 ′, including primer sequences: P2-PCR, 5′ -AATGATACGGCGACCACCGA-3 ′), recovery; (D) PCR: 5-10 rounds of amplification target sequence (enrichment and sequencing primers for P1-PCR\P2-PCR).

### Sequencing, raw data processing and SNP genotyping

Illumina Solexa sequencing was done by sequencer Hiseq 2000, and sequencing type was SE50. We make sure that clean data were at least more than 0.6 G (0. 8 × C) for each individual. After sequencing, the raw data were processed in three steps: first, we allocated the raw data to its own origin sample according to the labels (4 ~ 8bp) used for sequencing to distinguish samples (4 ~ 8bp); secondly, we filtered label sequences and the joint to exclude the pollution; thirdly, we discard those raw data with low quality base (Q ≤ 5 (E)) number accounted for more than half of the whole reads.

The rest clean and high quality raw data were used for further SNPs calling by software Stacks (http://catchenlab.life.illinois.edu/stacks). Software Stacks uses short-read sequence data to identify and genotype loci in a set of individuals either de novo or by comparing to a reference genome. From reduced representation Illumina sequence data, such as RAD-tags, Stacks can recover thousands of single nucleotide polymorphism (SNP) markers useful for the genetic analysis of crosses or populations [70]. We used SPSS 24.0 (https://spss.en.softonic.com/) to execute the significance test of difference in average frequency of total SNPs, homozygous SNPs and heterozygous SNPs between wild and cultivated loquat by using *T* test of independent samples. For further analysis, we filtered the genotype data with the loss rate less than 50% (Additional file 1: Table S1) or with no loss (Additional file 1: Table S2).

### Population genetics and evolutionary analysis

We used Arliquin 3.11 software [71] to execute the AMOVA and genetic differentiation analysis using SNP dataset with no loss/gap (Additional file 1: Table S2). Neighbor-Joining method [72, 73] was employed to construct the phylogenic relationships between loquat genotypes based on SNP dataset with no loss/gap (Additional file 1: Table S2) by using program MAGE 5.0 [74], and removed all the ambiguous positions for each sequence pair and set bootstrap = 500. The PCA analysis was executed by software EIGENSOFT 3.0 [75] using SNP dataset with the loss rate less than 50% (Additional file 1: Table S1). The software Structure v2.3.4 [76] was used to analyze the genetic structure using SNP dataset with the loss rate less than 50% (Additional file 1: Table S1) with admixture model, and K was set from 2 to 6.

## Additional file

Additional file 1:
**Table S1.** SNP dataset with less than 50% loss. **Table S2.** SNP dataset with no gap. (XLS 27844 kb)

## Abbreviations

PCA: Principal component analysis
SNP: Single nucleotide polymorphism
